# Associations of environmental factors with total cholesterol level of middle-aged and elderly people in China

**DOI:** 10.1186/s12889-022-14922-y

**Published:** 2022-12-23

**Authors:** Hao Li, Miao Ge, Zehua Pei, Jinwei He, Congxia Wang

**Affiliations:** 1 Institute of Healthy Geography, School of Geography and Tourism, Shaanxi Normal University, 620 West Chang’an Street, Chang’an District, Xi’an, 710119 China; 2 Medical School, Yan’an University, 580 Shengdi Road, Yan’an, 716000 China; 3 Department of Cardiology, the Second Affiliated Hospital of Medical College, Xi’an Jiaotong University, No. 157, Xiwu Road, Xi’an, 710004 China

**Keywords:** Cardiovascular disease, Total cholesterol level, Middle-aged and elderly people, Environmental factors, GeoDetector, Geographically weighted regression

## Abstract

**Background:**

Dyslipidemia is a key factor causing cardio cerebrovascular diseases, and the total cholesterol (TC) is an important lipid indicator among them. Studies have shown that environmental factors have a strong association with TC levels. Previous studies only focused on the seasonal variation of TC level and the short-term effects of some environmental factors on TC level over time, and few studies explored the geographical distribution of TC level and quantified the impact of environmental factors in space.

**Methods:**

Based on blood test data which was from China Health and Retirement Longitudinal Study (Charls) database, this study selected the TC level test data of middle-aged and elderly people in China in 2011 and 2015, and collected data from 665 meteorological stations and 1496 air pollutant monitoring stations in China. After pretreatment, the spatial distribution map of TC level was prepared and the regional statistics were made. GeoDetector and geographically weighted regression (GWR) were used to measure the relationship between environmental factors and TC level.

**Results:**

The TC level of middle-aged and elderly in China was higher in females than in males, and higher in urban areas than in rural areas, showing a clustered distribution. The high values were mainly in South China, Southwest China and North China. Temperature, humidity, PM_10_ and PM_2.5_ were significant environmental factors affecting TC level of middle-aged and elderly people. The impact of pollutants was more severe in northern China, and TC level in southern China was mainly affected by meteorological factors.

**Conclusions:**

There were gender and urban-rural differences in TC levels among the middle-aged and elderly population in China, showing aggregation in geographical distribution. Meteorological factors and air pollutants may be very important control factors, and their influencing mechanism needs further study.

## Background

Total cholesterol (TC) refers to the sum of the cholesterol contained in all lipoproteins in the blood. Cholesterol is essential for the formation of cholic acid and cell membranes. It is also necessary for hormone synthesis. In recent years, human dyslipidemia has attracted much attention as an independent and modifiable risk factor for cardiovascular and cerebrovascular diseases [1]. Cardiovascular and cerebrovascular diseases are the leading cause of death in the world. Such chronic diseases kill about 17 million people a year, accounting for about 30% of all deaths, according to the statistics of WHO. The incidence rate and mortality of cardiovascular and cerebrovascular diseases in Chinese population are still rising, bringing heavy medical burden to society and families. High levels of TC can affect normal blood circulation and may lead to local obstruction. Ischaemia following obstruction hypoxia, many adverse effects occur and is also a cause of cardio cerebrovascular disease in some individuals [2].

Many physiological indicators in the human body have very distinct rhythm characteristics, which is also confirmed by many studies in various regions of the world [3]. A temporally seasonal pattern of change has similarly been found in studies on lipid levels [4–6], so some scholars speculated whether this rule is the cause of seasonal changes in cardiovascular and cerebrovascular diseases [7]. Such studies are numerous but still suffer from significant drawbacks. For example, the study areas differed, and there was a large variation in their results. Each season has obviously different climate characteristics. At present, the relationship between various meteorological factors such as temperature, humidity, precipitation, air pressure and TC level of human body is not clear, and which risk factors have a stronger effect still needs to be studied.

Studies on the impact of air pollutants on total cholesterol level are frequently reported, which are mostly found in cohort studies in high-income countries, and the relationship between exposure to ambient air pollutants and lipid indexes has been discussed [8, 9]. Some studies have been conducted with specific populations such as adolescents, patients with chronic diseases responding to lipid indicators of pollution exposure [10], but the results were not consistent. Because of the unique geographical and environmental characteristics of the regions, TC levels in humans do not only show seasonal variation, but may also have spatial and geographic distribution patterns at the same time. At present, there are few reports on the geographical distribution of TC. In recent years, climate change continues to intensify and extreme weather appears more and more frequently. It is urgent to study the current climate characteristics and predict the possible impact of climate change on health indicators of residents in the future.

This study first explored the geographical distribution pattern of TC level of middle-aged and elderly people in China, and then quantified the associations and regional differences between environmental factors and TC level of middle-aged and elderly people based on GeoDetector and geographic weighted regression (GWR) method. This study is of great significance for public health policy-making.

## Methods

### Health data

**Fig. 1:**
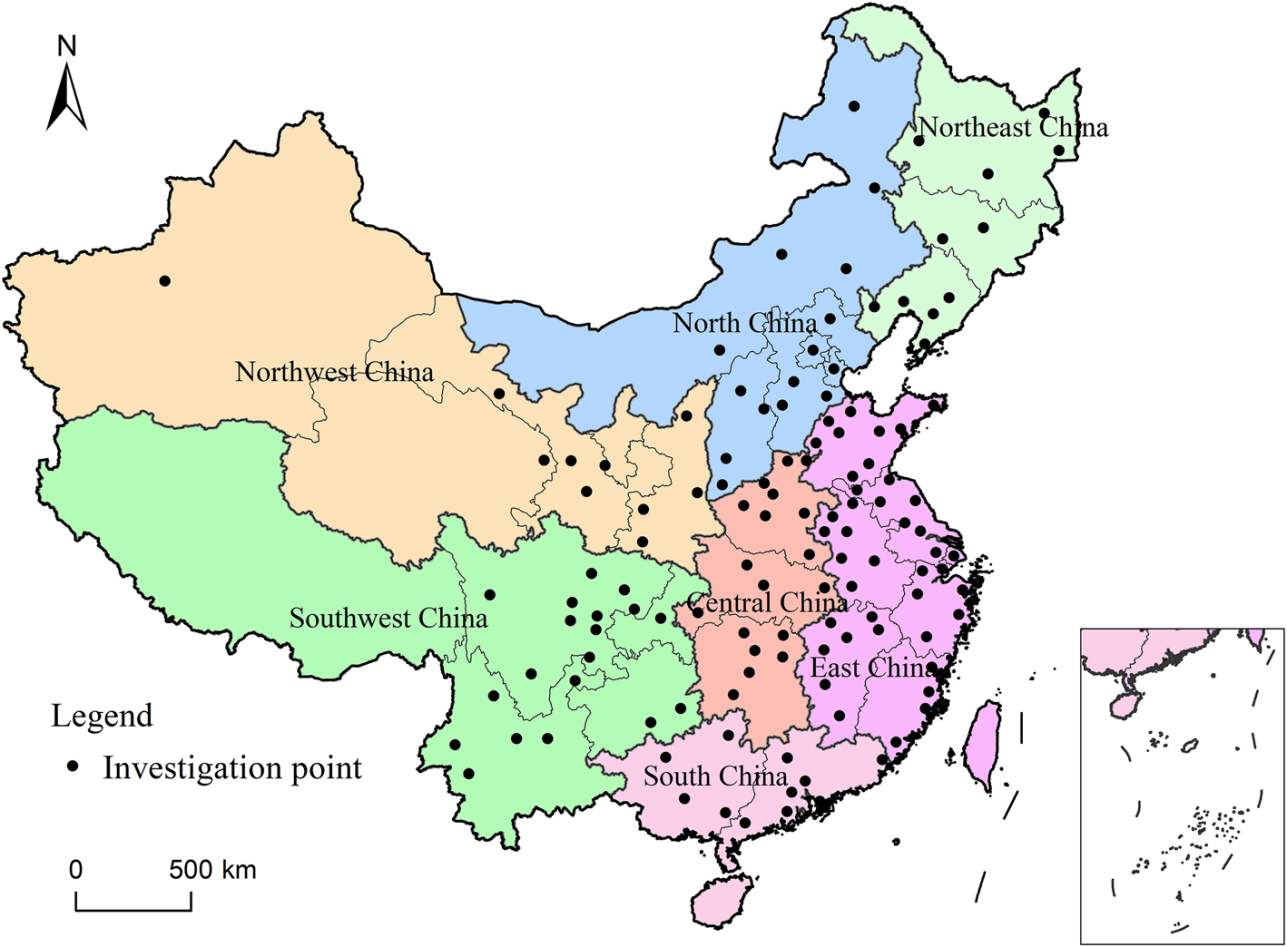
Investigation points and seven geographical regions

TC data of middle-aged and elderly people in China were obtained from China Health and Retirement Longitudinal Study (Charls) database of Peking University [11]. Charls data was designed to collect human health indicators representing middle-aged and elderly people over 45 years old in China to promote the study of population aging in China. The survey began in 2011 with coverage of 28 provinces, 150 prefectures, and 450 villages containing approximately 19,000 people. Blood test data are only available for 2011 and 2015. The data in 2011 were the baseline survey data, and the follow-up survey data in 2015 were supplemented appropriately. After pretreatment and elimination of specific and null values, a total of 13,354 TC level data covering 125 cities in China in 2015 were obtained (Fig. 1). The baseline data in 2011 was about 11,647. According to the personal information of each subject, the TC data in 2011 and 2015 were statistically differentiated by urban and rural areas, men and women, and the prefecture-level city region. We obtained the urban and rural differences of each person with a unique ID in Charles. Subjects belonging to urban and rural areas were distinguished according to their residential areas (villages or communities).

### Geographic environment data

The meteorological data came from the National Meteorological Science Data Center, which covers 665 meteorological monitoring stations in 31 provinces and autonomous regions across China. Data indicators include temperature, humidity, precipitation, sunshine hours, air pressure, wind speed and so on in 2011 and 2015. The air pollution data came from 1496 national ambient air quality monitoring stations released by China Environmental Monitoring Station, including six air pollution monitoring indicators including CO, NO_2_, SO_2_, O_3_, PM_2.5_ and PM_10_. As China’s ambient Air Quality standard was implemented in 2012, the corresponding air pollutant data of each city in 2011 were not obtained, and the data used in this study was from 2015. We eliminated the days with serious data loss through Python, and finally calculated the annual average value of environmental factors of each monitoring site. Ordinary Kriging interpolation in ArcGIS10.2 was used to interpolate data from meteorological stations and air pollution monitoring stations to cover the whole study area. All parameters are optimal by default. Finally, all environmental factors were statistically divided according to the boundaries of prefecture level cities to match the TC level data of subjects in each region.

### Moran index

Moran’s *I* was used to measure spatial interdependence between data and to characterize their spatial distribution types.

$$I=\frac{n\sum_{i=1}^n\sum_{j=1}^n{\omega}_{i,j}\left({y}_i-\overline{y}\right)\left({y}_j-\overline{y}\right)}{\left(\sum_{i=1}^n\sum_{j=1}^n{\omega}_{ij}\right)\sum_{i=1}^n{\left({y}_i-\overline{y}\right)}^2}$$

In the formula: *I* is the global Moran index, *n* is the total number of regions, *i, j* denote a city, *ω*_*ij*_ is the spatial weight, *y*_*i*_, *y*_*j*_ is the TC level, $\overline{y}$ is the average TC level.

Moran’s *I* index ranges from − 1 to 1, and *I* > 0 indicates positive spatial correlation. The larger the value, the higher the spatial agglomeration. *I* < 0 indicates negative spatial correlation. The smaller the value, the greater the spatial difference. *I* = 0 indicates spatial randomness.

### GeoDetector model

GeoDetector is a method to measure spatial stratification heterogeneity, which can effectively find the environmental driving factors that associated with human TC level. It is based on the assumption that if a factor is related to the TC of the human body, the geographical spatial distribution pattern of them should be similar. The advantage of the GeoDetector model is that it does not require linear characteristics of data and is not affected by collinearity between multi-variable data.

**Fig. 2:**
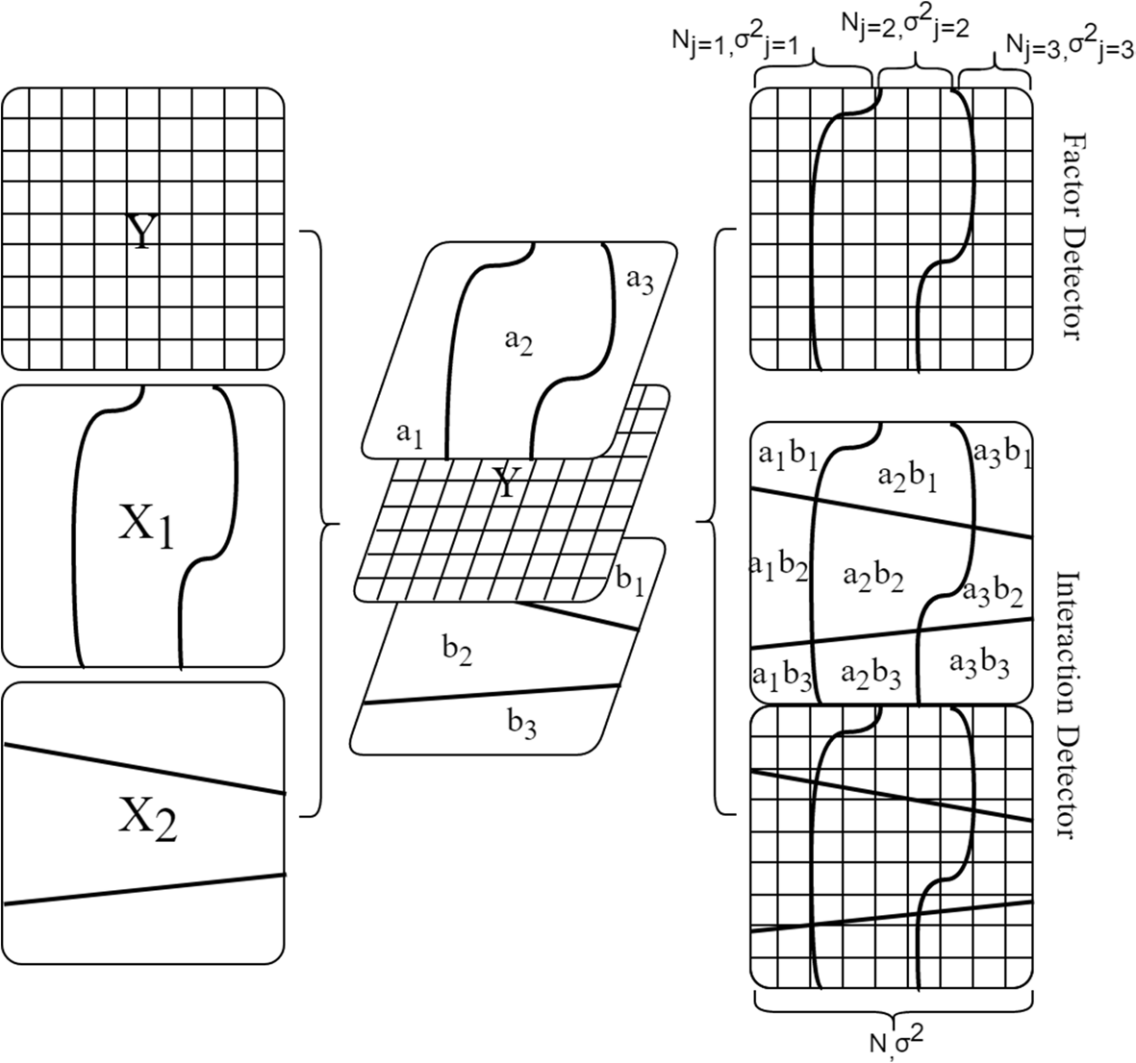
GeoDetector model (Y represents the geographic distribution of TC levels; X represents a certain environmental factor; a, b represent the classification of a certain environmental factor)

The two detection methods of GeoDetector model are shown in fig. 2. The input data of the GeoDetector model is required to be type variables, which represent the hierarchical information of variables. If the type variables are continuous variables, they need to be discretized. In this study, the “GD” package in R4.1.2 software (https://mirror.lzu.edu.cn/CRAN/) was used to build the GeoDetector model. The “corrplot” package was used for drawing, the classification interval of environmental factors was set to 3 ~ 8 categories, and the spatial discretization method used equal interval, geometric interval, natural discontinuity, quantile, standard deviation, etc. Finally, the results were compared to find the optimal model. The similarity of geographical spatial distribution pattern between TC and environmental factors was measured by *q* value (Factor Detector), which can be expressed as:

$$q=1-\frac{1}{N{\sigma}^2}\sum_{j=1}^L{N}_j{\sigma}_j^2$$

where *q* is a similarity between explanatory variables and geographic distribution of TB incidence, *j* = 1, 2... *L* is the number of categories; *N*_*j*_ and *N* are the number of layers *j* and the number of regional units; ${\sigma}_j^2$ and *σ*^2^ are *j* and the regional variance respectively. The range of *q* is [0,1]. The larger the *q* value, the greater the geographical distribution similarity between the explanatory variable and the dependent variable, indicating the greater the potential impact on the dependent variable.

In the correlation between actual environment and total cholesterol index, each environment factor not only has a separate effect, but also may have an interactive effect. The correlation between environment and TC not only has separate effect, but also may have an interactive effect. The GeoDetector model can measure the interaction between two factors (Interaction Detector *X*_*1*_, *X*_*2*_), and compare the *q* value between the interaction and the independent influence, and finally divide the interaction into 5 categories (Table 1).

**Table. 1:** The type of factor interaction expression

Formula	Interaction
*q*(*X*_*1*_, *X*_*2*_) < Min(*q*(*X*_*1*_),*q*(*X*_*2*_))	Weaken, nonlinear
Min(*q*(*X*_*1*_),*q*(*X*_*2*_*)*) < *q*(*X*_*1*_,*X*_*2*_) < Max(*q*(*X*_*1*_),*q*(*X*_*2*_))	Weaken, unilateral
*q*(*X*_*1*_, *X*_*2*_) > Max(*q*(*X*_*1*_),*q*(*X*_*2*_))	Enhance, bilinear
*q*(*X*_*1*_, *X*_*2*_) = *q*(*X*_*1*_) + *q*(*X*_*2*_)	Independent
*q*(*X*_*1*_, *X*_*2*_) > *q*(*X*_*1*_) + *q*(*X*_*2*_)	Enhance, nonlinear

### Geographically weighted regression (GWR) model

GWR model is an extension of the linear regression model. Different from the traditional global regression coefficient, the result is no longer a unified value, but a local regression coefficient obtained according to different geographic space division units. Its advantage is that it can quantify the influence of the independent variable on the dependent variable as the spatial location changes. The GWR equation is:

$${Y}_i={\beta}_0\left({u}_i,{v}_i\right)+\sum_{j=1}^p{\beta}_j\left({u}_i,{v}_i\right){X}_{ij}+{\varepsilon}_i$$

Where, Y is the TC level; X is the explanatory variable; β_j_(u_i_, v_i_) (j = 0, 1, ⋯, p) is the spatial weight function, p is the number of regression parameters, u_i_, v_i_ is the coordinate of sample point i, and ϵ is the random error.

The geographically weighted regression model was completed in ArcGIS 10.2 (https://www.esri.com/en-us/home). In order to avoid uneven data distribution, ADAPTIVE was selected as the kernel type. It can automatically select the spatial environment scale according to the element density function. AICc method was used to determine the optimal bandwidth. After the GWR model was established, the regression coefficients of various environmental factors were calculated.

## Results

### Descriptive analysis

Table 2 shows the statistics of 11,647 subjects in 2011 and 13,354 subjects in 2015 included in this study, and the statistical results are classified according to urban-rural differences and gender. It expressed the mean and standard deviation of TC of residents with different gender and with urban-rural difference, respectively. As can be seen from the table, the average TC level of the middle-aged and elderly in China was 192.959 mg/dl in 2011, which decreased to 183.710 mg/dl in 2015, with a decrease of 4.79%. The standard deviation of TC is large, indicating that there are large differences in TC levels in different regions. In 2011, the TC level of rural women was higher than that of urban women. In 2015, the TC level of all groups in urban areas was significantly higher than that in rural areas, which may be caused by the unreasonable dietary structure and sedentary lifestyle of urban residents. In terms of gender, the TC level of both men and women decreased significantly in the past 5 years and TC levels of males were significantly lower than those of females, with a difference of 5.02 and 6.07%, respectively. Except for middle-aged and elderly women in 2011, the urban-rural difference was roughly the same as that of the whole group, and the difference between urban and rural showed no impact on a particular gender. The TC level of women in both urban and rural areas was significantly higher than the average of the whole population, which was the main contributor to the high level of TC in China.

**Table 2:** Statistics of the subjects in 2011 and 2015

Classification	2011 Value	2015 Value
Body Mass Index (kg/m^2^, mean ± SD)	23.542 ± 3.903	23.977 ± 5.232
Age (mean ± SD)	59.201 ± 9.716	59.522 ± 10.543
Smoking status N (%)		
Never	7173 (61.6%)	8027 (60.1%)
Former	968 (8.3%)	1708 (12.8%)
Current	3536 (30.1%)	3619 (27.1%)
Drinking frequency N (%)		
Never	7186(61.7%)	8599 (64.4%)
1/month	884(7.6%)	1162 (8.7%)
1/month	3569(30.7%)	3593 (26.9%)
Total cholesterol Value (mean ± SD, mg/dl)		
Total	192.959 ± 38.893 (*N* = 11,647)	183.710 ± 36.556 (*N* = 13,354)
Urban	193.741 ± 38.917 (*N* = 4277)	185.639 ± 36.570 (*N* = 5082)
Rural	192.506 ± 38.899 (*N* = 7370)	182.525 ± 36.558 (*N* = 8272)
Male	187.652 ± 38.917 (*N* = 5413)	177.526 ± 36.557 (*N* = 6150)
Urban	189.181 ± 38.917 (*N* = 1944)	179.857 ± 36.571 (*N* = 2291)
Rural	186.795 ± 38.900 (*N* = 3469)	176.142 ± 36.559 (*N* = 3859)
Female	197.568 ± 38.895 (*N* = 6234)	188.989 ± 36.557 (*N* = 7204)
Urban	197.541 ± 38.918 (*N* = 2333)	190.386 ± 36.571 (*N* = 2791)
Rural	197.584 ± 38.899 (*N* = 3901)	188.106 ± 36.558 (*N* = 4413)

*SD* Standard Deviation, *N* Number of individuals.

### Spatial patterns of the TC level of middle-aged and elderly

**Fig. 3:**
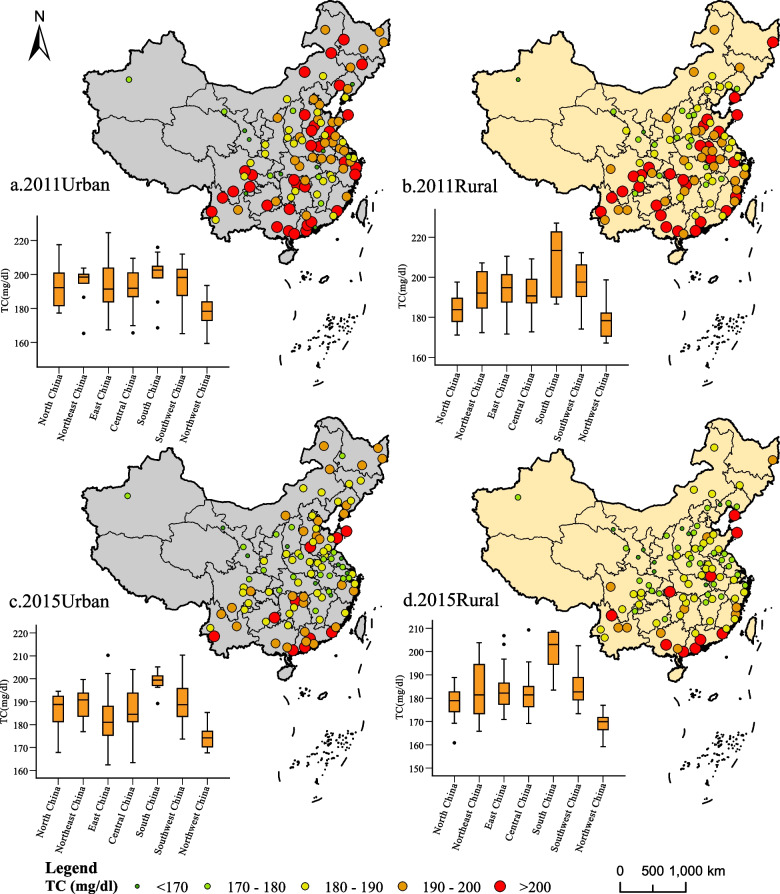
Spatial distribution and regional statistics of TC level in urban and rural middle-aged and elderly in 2011 and 2015

Figure 3 shows the spatial distribution of TC level of middle-aged and elderly people in urban and rural areas in 2011 and 2015. Moran’s *I* was used to measure spatial aggregation characteristics. It can be seen that the TC of the middle-aged and elderly population in urban areas was at a high level in 2011, and the Moran index showed a random distribution feature (Table 3). In 2011 and 2015, TC levels of middle-aged and elderly population in rural and urban areas showed a trend of aggregation, and Moran index showed that the aggregation was increasing.

**Table 3:** The results of Moran index

	Moran’s *I*	Z score	*P* value	Pattern
2011Urban	0.006	1.002	0.317	Random
2011Rural	0.073	5.451	0	Clustered
2015Urban	0.132	9.181	0	Clustered
2015Rural	0.108	7.864	0	Clustered

In order to explore the aggregation areas with high or low TC level distribution, the country was divided into seven geographical regions for regional statistics according to the geographical and climatic characteristics of the country (Fig. 1). Figure 3 shows the comparison of TC levels in each geographical region and the overall distribution of TC. In the past 5 years, the TC level of the middle-aged and elderly showed a decreasing trend in all regions. The high value mainly concentrated in South China and Southwest China, while the TC level was lower in Northwest China. The difference was more obvious in rural than in urban. Figure 3a and c show that North China and East China are at the same level. However, Shandong province was higher than other provinces in East China. Most of Shandong province was located in the North China Plain. According to the distribution map, urban areas in the North China Plain should also be a wide hot spot area.

### Results from GeoDetector

Table 4 shows the *q* value between each environment factor and TC level and the classification results of each factor. The results show that there were 4 items in urban areas passed the significance test among the environmental factors. The geographical distribution of PM_10_ and TC had the greatest similarity (0.282). Both inhalable particulates showed a potential correlation with TC levels. The geographical distribution of temperature and humidity in meteorological factors showed obvious similarity with that of TC. The order of *q* value was PM_10_ > temperature > humidity > PM_2.5_. There were 8 items in rural areas passed the significance test. PM_10_ (0.397) and air temperature (0.368) had the greatest similarity with the geographical distribution of TC, indicating that there was no difference between urban and rural areas in the impact of the two factors on TC. The common environmental factors affecting the spatial distribution of TC in urban and rural areas were temperature, humidity, PM_10_ and PM_2.5_. Compared with urban areas, air pollutants NO_2_, SO_2_, CO showed similar spatial distribution of TC in rural areas, indicating that rural residents may be more sensitive to changes in air pollutant concentration. The *q* values in rural areas are in the order of PM_10_ > temperature > NO_2_ > PM_2.5_ > SO_2_ > CO > precipitation > humidity.

**Table 4:** Factor detection results and classification

Factors	2015Urban			2015Rural		
	*q*	*P* value	Class	*q*	*P* value	Class
Pressure	0.175	0.057	7	0.083	0.534	7
Sunshine hours	0.092	0.127	5	0.095	0.249	7
Humidity	0.274^a^	0	7	0.173^a^	0.017	6
Temperature	0.278^a^	0.001	8	0.368^a^	0.001	8
Precipitation	0.126	0.118	7	0.176^a^	0.018	7
Wind Speed	0.173	0.097	8	0.122	0.48	7
CO	0.067	0.509	8	0.213^a^	0.009	8
NO_2_	0.169	0.155	8	0.323^a^	0.001	6
O_3_	0.138	0.09	8	0.156	0.089	8
PM_10_	0.282^a^	0	6	0.397^a^	0	8
PM_2.5_	0.261^a^	0	8	0.309^a^	0.002	8
SO_2_	0.144	0.319	8	0.258^a^	0.01	8

^a^The association was significant

**Fig. 4:**
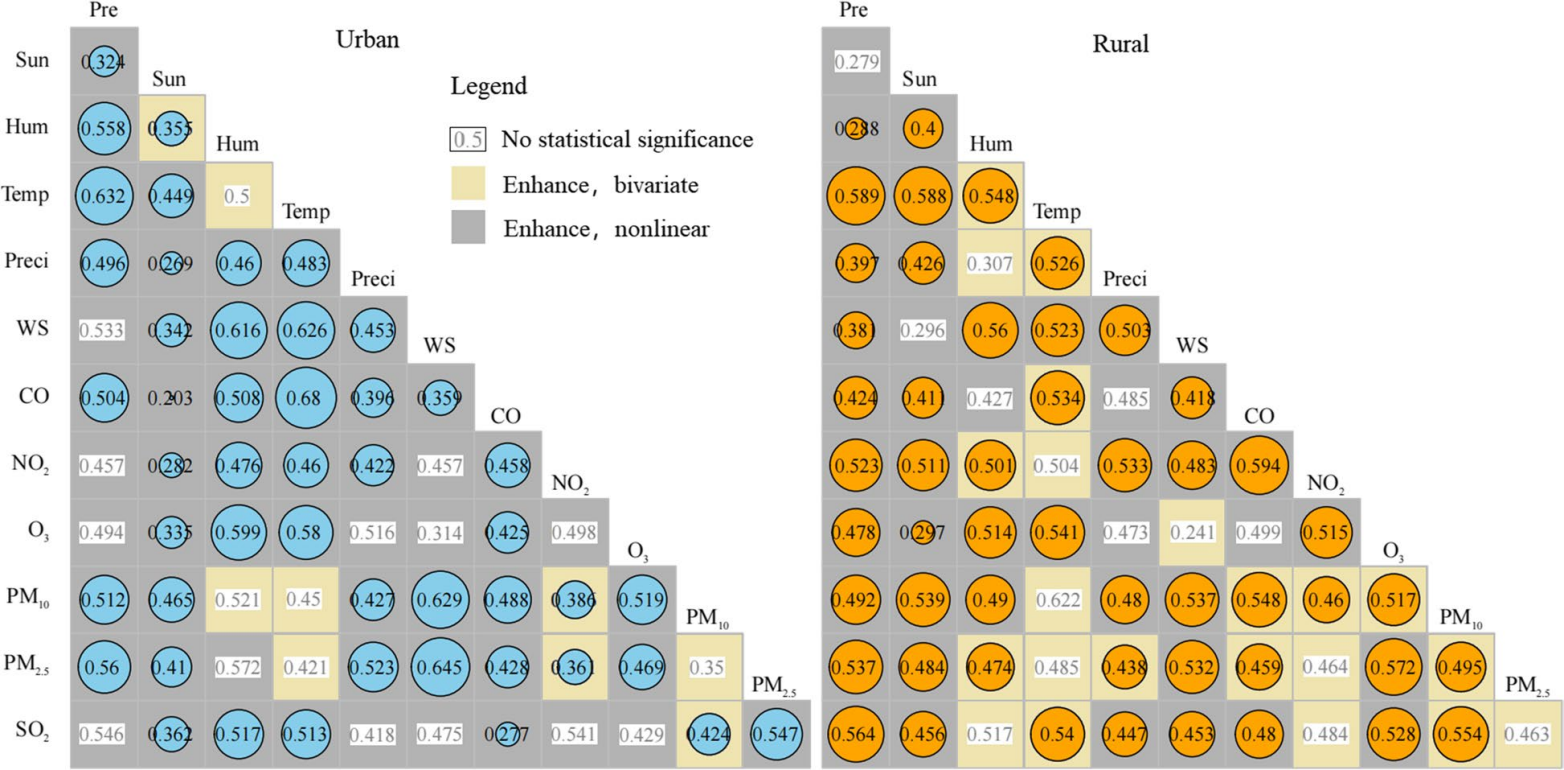
Interaction detection results (Pre: Pressure, Sun: Sunshine hours, Hum: Humidity, Temp: Temperature, Preci: Precipitation, WS: Wind Speed)

The results of interaction detection (Fig. 4) show that the combination of any two environmental factors plays an important role in the potential impact on TC level, and the impact was greater than that of single factor. After excluding insignificant factors by student’s t test, it can be seen that the *q* value of most interactions was greater than 0.4. Compared with that of single factor, the interactions showed an enhancement effect. According to Table 1, the interaction type of most factors was nonlinear enhancement. The factors with the greatest interaction intensity in rural areas was CO and NO_2_ (0.594), followed by temperature and pressure (0.589), indicating that rural areas with significant different characteristics of air temperature, pressure, CO and NO_2_ need special attention. The factor with the greatest interaction intensity in urban areas was temperature and CO (0.68), followed by wind speed and PM_2.5_ (0.645). The type of interaction between most environmental factors was nonlinear enhancement, which means that the synergy between environmental factors may be more complex than the interaction between the two factors.

### Results from geographically weighted regression

GeoDetector can find out potential environmental factors that associated with TC, but it cannot determine the positive or negative impacts of this factor. In this study, GWR method was used to quantify the positive or negative impact of a single environmental factor in different regions on TC by using four environmental factors (temperature, humidity, PM_10_, PM_2.5_) that have impacts on both urban and rural areas.

**Fig. 5:**
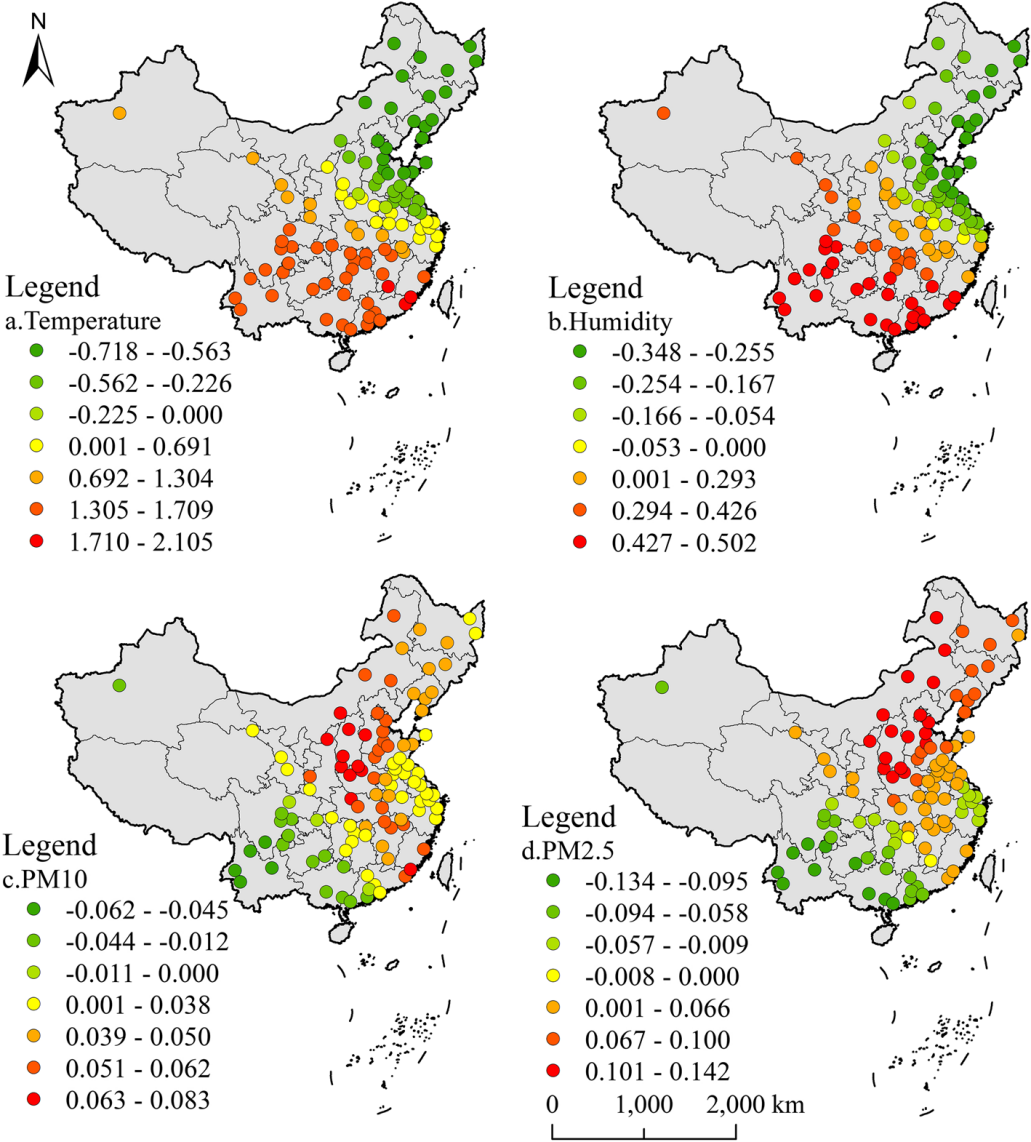
GWR results of urban TC level and environmental factors in 2015

The GWR fitting R^2^ of urban areas was 0.316 (Fig. 5). The results showed that there was a significant north-south difference in the impact of temperature on TC level in urban areas. There was a positive correlation in the south. And TC level in some regions of Guangdong Province was more affected by temperature. From southwest to northeast, the impact of temperature showed a decreasing trend, and showed a negative impact in north China after roughly crossing the north-south demarcation line. The correlation between humidity and TC had a very similar distribution trend compared with that of air temperature. However, the regression coefficient of humidity was smaller than that of temperature, indicating that air temperature was the most important meteorological factor affecting TC in urban areas.

The regression coefficients of the two inhalable particles in air pollutants also had a similar distribution trend. The correlation was positive in North China and Northeast China, decreasing from north to south, and negative in East China and Southwest China. The influence of PM_2.5_ on urban TC showed negative correlation in Zhejiang Province. Comparing the regression coefficients of the two inhalable particles, it can be seen that PM_2.5_ had a stronger impact. Urban areas in North China and Northeast China were more polluted areas. Research showed that long-term exposure to serious air pollutants may lead to the rise of TC level. The air quality in South China and Southwest China was better, and the impact of pollution on TC was small, which may be the reason for the negative correlation in Southern urban areas.

**Fig. 6:**
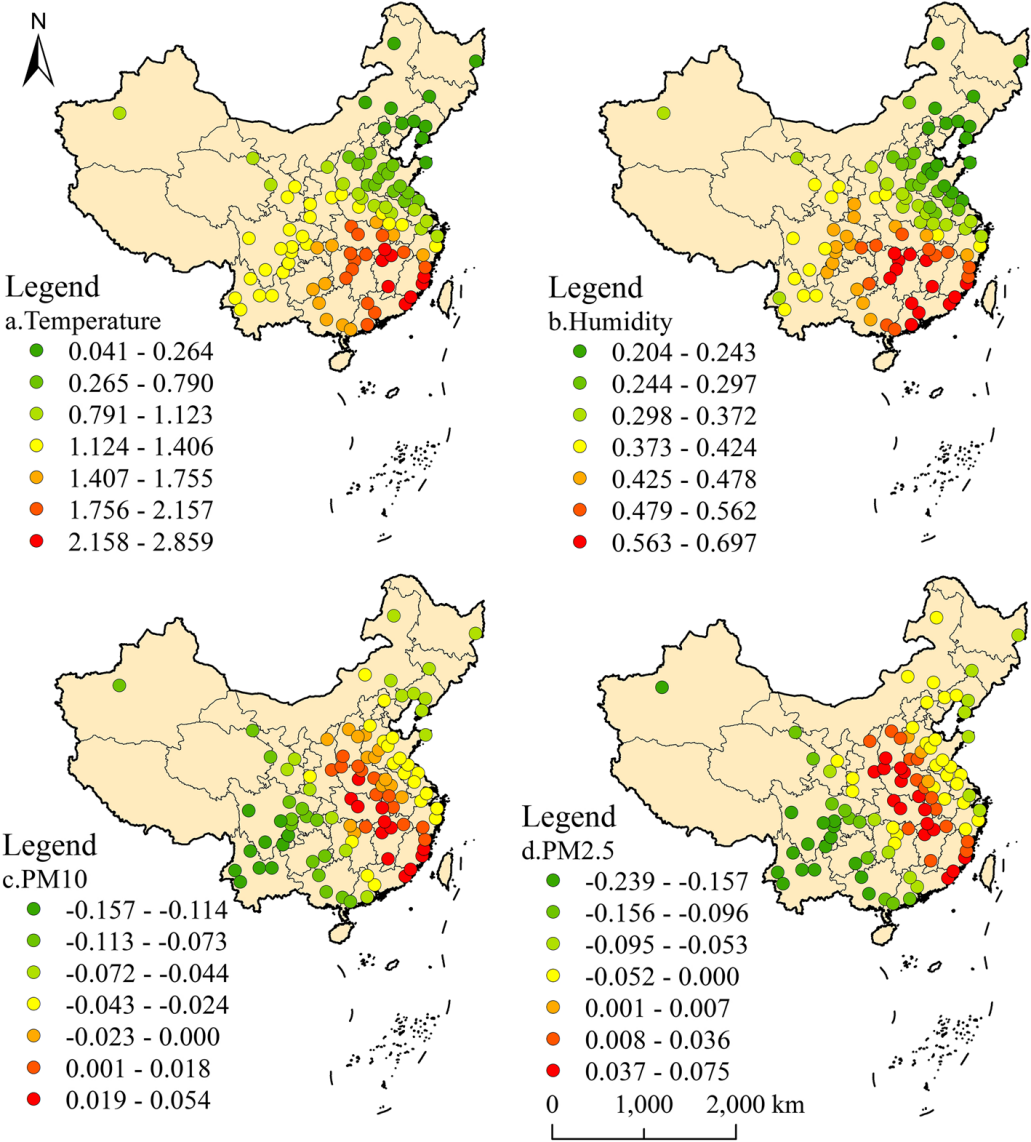
GWR results of rural TC level and environmental factors in 2015

The regression coefficient in rural area was obviously different from that in urban area (Fig. 6), and the GWR fitting R^2^ of rural area was 0.369. There was a positive correlation between temperature and humidity on TC level in rural areas. Contrary regression coefficients showed that although the distribution trend was similar, temperature and humidity showed different correlation with TC level in urban and rural areas in northern China. The region with the strongest response to temperature and humidity was located in South China, and then the influence degree decreased from southwest to northeast.

The impact of two inhalable particulates on TC level in rural areas was completely different from that in cities. The impact effects of PM_10_ and PM_2.5_ were positive correlation in central and southern China, and gradually decreased to the northeast and west. Inhalable particulate matter had the strongest impact on urban TC in North China. However, in rural areas of North China, the impact of two kinds of inhalable particulate matter was close to 0.

## Discussion

Even though the TC level of Chinese residents had shown a significant increase since the 21st century, the TC level survey results of both men and women in 2015 were still lower than that in 2011. At the same time point, the TC level of middle-aged and elderly women was significantly higher than that of men, which may be related to the changes of estrogen level in middle-aged and elderly women during menopause. Some studies had confirmed that estrogen plays a positive role in maintaining a low level of TC. And the loss of the protective effect of estrogen will significantly increase the obesity rate and the risk of dyslipidemia in middle-aged and elderly women [12–14]. This indicated that more attention should be paid to the lipids level in this group people. The distribution of TC level in urban areas was higher than that in rural areas. With the increasing speed of urbanization and industrialization, the lifestyle of Chinese residents had changed greatly. More people travel by motor vehicles rather than by bicycle or on foot, and sedentary lifestyle has become the main way of life. Reduced exercise intensity will increase the biological aging rate of middle-aged and elderly people, while lack of activity may lead to obesity [15]. Studies showed that the overall physical activity of middle-aged and elderly urban population was lower than that of rural population, which may not be conducive to the reduction of individual TC level [16].

Combined with GeoDetector model and GWR method, this study attempted to find potential environmental drivers that affect the geographical distribution of TC levels among middle-aged and elderly people in China and quantified their relationships. GeoDetector model was formally proposed by Wang and Xu in 2017 [18]. Prior to this, GeoDetector model had been successfully applied in public health fields such as neonatal neural tube malformation [19] and hand, foot and mouth disease [20]. Combined with related medical and toxicological microscopic studies, it can be confirmed that GeoDetector model has a very wide range of applicability. GWR method was also used to measure the influence of complex factors on human health index or disease, which can make up for the disadvantage that the nonlinear effect analysis of GeoDetector cannot determine whether the relationship between the two was positive or negative.

For a long time, TC level of the middle-aged and elderly population had shown seasonal variation, and most studies attributed the reason to climate, but did not explore the relationship between a single factor and TC level. In recent years, only a few cohort studies had been conducted on the temporal correlation between meteorological factors and blood lipids level [3, 21]. This study found that two meteorological factors were associated with TC level of the middle-aged and elderly population, based on GeoDetector results. Temperature had a very similar geographical distribution with TC level. This study found that after urban and rural classification, South China showed a positive correlation, while North China showed a negative correlation. Temperature in rural areas showed a positive correlation with TC, although the fitting R^2^ was not high. Zheng et al. found that the relationship between outdoor temperature and serum TC level was non-linear, and there was a significant negative correlation between temperature and TC in the thermal effect, while there was no significant correlation between them in the cold effect [22]. Halonen et al. found that there was no statistically significant change in TC when the temperature increased by 5 °C [23]. Basu et al. ‘s cohort study on middle-aged women found that temperature changes were always negatively correlated with TC in warm or cold seasons [24]. This result was different from those of this study. According to the comparison, most studies were based on time series and did not explore the differences in space. In a regional study in China, similar conclusions were obtained [25]. Regional differences may make the associations between temperature and TC different from that of time series. A 5-year longitudinal study in China showed that temperature was an independent risk factor for lipid levels [26]. However, this study explored the interaction between geographical factors. And the results showed that the temperature interacted with almost all other environmental factors.

Humidity was also an important factor affecting TC level. Similar to temperature, in urban areas, humidity and TC level showed a positive correlation in the south, but negative in the north. In rural areas, humidity was positively correlated, and the influence intensity was low in northern China. Humidity was related to physiological responses through heat stress and hydration [27]. The humidity characteristics of an area may have affected the local diet culture for a long time, thus indirectly affecting the TC level of the human body. Juna et al. found that humidity was positively correlated with metabolic syndrome in human body, and high humidity may increase the risk of dyslipidemia [28]. Humidity was related to many metabolic indicators of human body, but many research results were inconsistent, and humidity was often removed as a confounding factor affecting model accuracy [29].

In recent years, the problem of urban air pollution had troubled many countries, and air pollutants had also caused a huge impact on human health indicators [30]. In this study, it was found that the geographical distribution of TC level was significantly correlated with PM_10_ and PM_2.5_. In urban areas, inhalable particulate matter in North China and Northeast China had a positive effect on TC level. Although air quality had improved significantly, the air pollutants were still higher in North China and Northeast China than in other regions. All but O_3_ pollutants (NO_2_, SO_2_, CO) in rural areas showed associations with TC. The pollution situation was better in rural areas relative to urban areas, so residents may be more sensitive to the change of gas pollutants. Urban areas had many sources of complex types of pollutants other than in rural areas, such as excessive car exhaust and plant emissions, so some of the pollutant effects may not be significant. A study on environmental air pollution and blood lipid levels in Shijiazhuang showed that air pollutants would significantly increase TC levels. And the elderly were more susceptible to air pollutants. After controlling the influence of gas pollutants, it was found that the influence of inhalable particulate matter becomes stronger, indicating that inhalable particulate matter was the main influencing factor [31]. Many studies were consistent with the research results of this study [32, 33]. Toxicological studies had shown that inhalable particles may cause inflammatory response and oxidative stress response and induce hepatocyte lipid degeneration [34, 35]. Results in South China and Southwest China had found negative correlation regression coefficients. Other studies had found that most time series studies showed positive correlation [36, 37], and only some short-term exposures showed negative correlation. These studies attributed the reason to confounding factors of short-term diet [38]. South China and Southwest China had less pollution and residents had not been exposed to air pollutants for a long time. The effects of pollutants were not a major factor.

This study found that environmental factors may also play a role in promoting. Research showed that meteorological factors such as humidity will increase the moisture absorption of aerosol particles, which will change the radiation intensity and lead to a great threat to human health. The classification of interaction showed that the promotion of environmental factors belongs to the category of nonlinear enhancement, and the nonlinear interaction of environmental factors in urban areas was obviously stronger than that in rural areas. Air pollutants in urban areas were more diverse and may have special and stronger human health impacts. The nonlinear enhancement effect illustrated that environmental factors have complex influencing mechanisms on TC levels and were not limited to the cooperation of both factors. These affecting mechanisms are not clear at present, and may exist in complex biological processes that still require further investigation.

This study has some advantages. Firstly, this is the first study to study the relationship between environmental factors and TC level in space based on GeoDetector model and GWR method. Secondly, GeoDetector method was used to conduct nonlinear research, avoiding the problem that data cannot be measured and collinear due to complex influences [39]. Thirdly, the data type of this study is comprehensive, and the middle-aged and elderly population is more important, which can provide valuable reference for policy making. The study also has some limitations. Firstly, GPS information of each subject was not obtained in the data, so some confounding factors were not considered, which may lead to a small range of errors when studying the correlation of environmental factors. Exploring the relationship between environmental factors and TC level on the basis of spatial distribution rather than time series may lead to neglecting short-term effects, and may lead to errors that are difficult to eliminate due to the huge differences in complex factors such as climate and economy between regions [40].

## Conclusions

There were gender and urban-rural differences in TC levels among the middle-aged and elderly population in China, and the TC levels showed a clustered distribution in South China, Southwest China and North China. Temperature, humidity, PM_10_ and PM_2.5_ had a significant impact on residents’ TC level. However, the influence among them was still unclear and needs further study.

## Data Availability

The datasets generated and/or analysed during the current study available in the Charls Study at https://charls.charlsdata.com/pages/Data/2015-charls-wave4/en.html. The environmental factors data were obtained from the China Meteorological Data Sharing Service System and Environmental Monitoring of China. The data acquisition requires application. However, the datasets used and/or analyzed during the current study are available from the corresponding author on reasonable request.

## Abbreviations

TC: total cholesterol
WHO: World Health Organization
Charls: China Health and Retirement Longitudinal Study
GWR: Geographically Weighted Regression
GPS: Global Positioning System
SD: Standard Deviation
CO: Carbon monoxide
O_3_: Ozone
NO_2_: Nitrogen dioxide
SO_2_: Sulfur dioxide
PM_10_: Particulate matter with particle size below 10 μm
PM_2.5_: Particulate matter with particle size below 2.5 μm
